# Unsigned surprise but not reward magnitude modulates the integration of motor elements during actions

**DOI:** 10.1038/s41598-023-32508-5

**Published:** 2023-04-03

**Authors:** Roula Jamous, Adam Takacs, Christian Frings, Alexander Münchau, Moritz Mückschel, Christian Beste

**Affiliations:** 1 Cognitive Neurophysiology, Department of Child and Adolescent Psychiatry, Faculty of Medicine, TU Dresden, Fetscherstrasse 74, 01307 Dresden, Germany; 2 University Neuropsychology Centre, Faculty of Medicine, TU Dresden, Dresden, Germany; 3 Cognitive Psychology Unit, Chair of General Psychology and Methodology, Faculty I — Psychology, University of Trier, Trier, Germany; 4 Institute of Systems Motor Science, University of Lübeck, Lübeck, Germany

**Keywords:** Human behaviour, Cognitive neuroscience, Cognitive control

## Abstract

It seems natural that motor responses unfold smoothly and that we are able to easily concatenate different components of movements to achieve goal-directed actions. Theoretical frameworks suggest that different motor features have to be bound to each other to achieve a coherent action. Yet, the nature of the “glue” (i.e., bindings) between elements constituting a motor sequence and enabling a smooth unfolding of motor acts is not well understood. We examined in how far motor feature bindings are affected by reward magnitude or the effects of an unsigned surprise signal. We show that the consistency of action file binding strength is modulated by unsigned surprise, but not by reward magnitude. On a conceptual and theoretical level, the results provide links between frameworks, which have until now not been brought into connection. In particular, theoretical accounts stating that only the unexpectedness (surprisingness) is essential for action control are connected to meta-control accounts of human action control.

## Introduction

It seems natural that motor responses unfold smoothly and that we are able to easily concatenate different components of movements to achieve goal-directed actions. For that, different features constituting a motor response or sequence need to become integrated^1,2^, just as for a coherent/holistic perception of our environment^3,4^. Different features defining an action (e.g. which hand/finger is to be moved) have to be “bound” to each other to achieve a coherent movement/response^1^. Even though recent years have seen progress in the understanding of the neurophysiological underpinnings of these motor feature binding processes^2,5–10^, the nature of the “glue” (i.e. bindings) between elements constituting a motor sequence and enabling a smooth unfolding of motor acts is not well understood.

Especially considering that "bindings" have been conceptualized as associations between action features^1^, this is astonishing because reward and reinforcement learning related processes are central to build and (re −)shape associations^11^. An in-depth investigation of how reward and reinforcement learning principles shape bindings between different features of motor responses, will provide insights into the nature of bindings. Likewise, this will inform how theoretical frameworks detailing how smooth motor planning and responding proceeds, can be connected to theories framing effects of reward. The effects of reward have been conceptualized in reinforcement learning accounts. The latter mostly refer to the processing of reward prediction errors (RPEs)^12^, which are signed quantities describing the difference between expected and received rewards^13,14^. Anticipation of reward fosters behavioral performance and the size of a potential reward scales with dopamine system activity carrying reward information^15,16^. Sometimes, however, behavior is not affected by the reward magnitude or the valence of a predicted event. Instead, the mere un-expectedness of non-occurrences of predicted events, no matter whether these are “good” or “bad” events, has an effect^12,17^. Such surprises provide a signal similar to an unsigned (valence-neutral) prediction error in reinforcement learning^12^, leading to the notion that violations of predictions (i.e. the surprisingness) and not necessarily the valence of rewards shape behavior. Thus, the question arises whether bindings between features determining motor sequences are affected by the magnitude and valence of rewards and/or by the surprisingness of predicted events, independent of the valence?

A consequence of bindings between features constituting a motor response is that once features for specific responses are activated, they are hardly available for the planning of other motor responses partly depending on the same features until the initiated motor response has been fully executed^2,18,19^. Therefore, an overlap between a to-be-performed and a planned response impairs performance, which is reflected by increased reaction times (RTs) and higher error rates^1,2,7,8,20^. The degree of performance impairment depends on the strength of binding^2,20–22^. The employed experimental approach therefore uses an “ABBA design”, which requires that a planned response (A) is executed only after another response (B) is planned and performed immediately. Performance of this immediate response (B) is better when there is no feature overlap between the A and the B motor response, compared to when features overlapped between both responses^2,9,20^. The strength of bindings between features constituting response A determines how much response performance on B declines in the feature overlap, compared to the non-overlap condition. We used an orthogonal manipulation of reward magnitude and surprisingness of a specific reward magnitude (i.e. unsigned reward prediction error^23^) across different experimental groups (see methods section). Participants were rewarded upon fast responses on stimulus B. If reward magnitude modulates bindings between action features, the modulation of the action feature binding effect should be higher with higher rewards and not modulated by the surprisingness of the reward magnitude. If, however, it is the un-expectedness/non-occurrences of predicted events that is most important, binding effects should be modulated in an identical way no matter whether the magnitude of reward is higher or lower than expected. Considering behavioral performance, we not only examine mean changes in binding strengths, but also in how far the consistency in the strength of action feature bindings are modulated. This is relevant since enhanced dopamine system signaling, as induced by rewards and surprising events^14^, decreases the intra-individual variability in behavior and neurophysiological correlates thereof^24–27^. It is, therefore, well-conceivable that effects of reward magnitude and/or surprise do not only modulate the degree of binding strength between action features. Instead, it may be the variability in the strength of binding that is decreased by effects of reward magnitude and/or surprise.

## Material and methods

### Participants

Four samples of participants were examined (first sample: *N* = 39, 18 female, *M*_age_ = 25.7, *SD*_age_ = 5.9; second sample: *N* = 39, 23 female, *M*_age_ = 25.9, *SD*_age_ = 3.8; third sample: *N* = 34, 19 female, *M*_age_ = 27.7, *SD*_age_ = 8.1; fourth sample: *N* = 16, 9 female, *M*_age_ = 26.9, *SD*_age_ = 4.6). Sample sizes corresponded to previous studies suggesting at least middle effect sizes for these kind of bindings^5,28^; thus we can be assured that given α = 0.05, 1-β was > 0.80. Based on self-reports, none of the participants had a history of psychiatric or neurological disorders and did not receive centrally acting medication. All participants had normal or corrected-to-normal vision and stated to be right-handed.

### Ethics statement

Before the experiment started, participants were informed about the procedures including data collection and publication. All participants provided written informed consent. After the experiment, participants received financial compensation of 15€, plus a bonus remuneration corresponding to their performance (see 2.4 Reward magnitude and surprise manipulations). The study was approved by the Ethics Committee of the TU Dresden and was conducted according to their guidelines, the tenets of the Declaration of Helsinki, and all relevant local regulations.

### Experimental task

**Figure 1:**
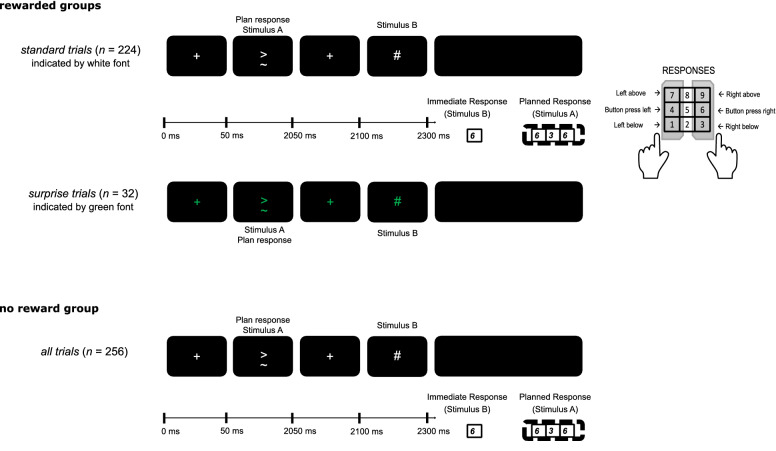
Schematic illustration of the R-R task. Stimulus and event timing as well as the response layout is depicted. Participants responded by using the numerical pad on a keyboard. The buttons of “1”, “4” and “7” were used for left responses, and “3”, “6” and “9” for right responses. Participants were instructed to use their left index finger on the left side of the pad, and their right index finger on the right side of the pad. For standard trials, stimuli are presented with white font color, green color was used for surprise trials (only rewarded group, top). In the no reward group (bottom), white font color was used.

To investigate motor feature integration (binding), we chose an established paradigm^2,20^, also known as R-R task, which is shown in Fig. 1.

This task was already used to in the context of EEG recordings^5,28^. The participants were seated at a distance of 60 cm in front of a 17-inch CRT display. For every trial, participants had to perform task A and task B in an interleaved ABBA sequence. Task B was embedded in task A. That is, participants were instructed to withhold the execution of a response to stimulus A until after presentation of and immediate response to stimulus B. Therefore, the effect of an already formed action plan (of task A) on the efficiency to plan and execute another action (of task B) can be observed. Every trial started with a fixation cross, which was displayed for 50 ms. This was followed by Stimulus A that was presented for 2000 ms. Stimulus A consisted of a left- or right-pointing arrowhead and a “ ~ ” either above or below the arrowhead. After stimulus A, another fixation cross was displayed again for 50 ms. Next, Stimulus B displayed either a “&” or a “#” for 200 ms. Following every trial, participants received a feedback about respective received or non-received points, which was displayed for 500 ms.

The response to stimulus A consisted of a sequence of three consecutive parts: first, a button press for the direction of the arrowhead (left or right center button), second, a button press for the position of the “ ~ ” (above or below the first button press), last, a button press again for the arrowhead direction (left or right center button, i.e. repeating the first button press). This response chain needed to be withhold until Response B was executed. To Stimulus B, participants had to respond immediately with either a left button press for “&”, or a right button press for “#”. After responding to stimulus B, a question mark indicates to now carry out the previously planned response to stimulus A. Since the sides of the correct button press for responses to task A and task B could be the same or different, this resulted in two conditions for the trials: feature overlap (same side of response for task A and task B) and no feature overlap (sides switched between responses for task A and task B). In the following, we will use the term “Immediate Response” for the response to task B, and the term “Planned Responses” for the three consecutive responses to task A. Even though participants were instructed to respond as fast as possible, there was no time limit for responding to either task A or B. To exclude reaction time outliers, only trials with an Immediate Response faster than 1500 ms were included in the analyses. To quantify response time for the Planned Responses, the accumulated time between the onset of the Immediate Response and the third button press of the response sequence is used. One experimental session consisted of 12 practice trials, and 256 experimental trials. Feature overlap and no feature overlap conditions occurred equally often. Every participant had at least 200 valid trials (i.e., first response within 1500 ms) with at least 100 of these trials in the feature overlap condition.

### Reward magnitude and surprise manipulations

For an orthogonal manipulation of reward magnitude and reward expectation (surprise) on action feature integration (binding), the R-R task was expanded. Participants were instructed to earn as many points as possible to increase their bonus for participating in the study. These points could be collected in every trial, based on the accuracy of the responses. Only trials with a correct Immediate Response were rewarded. However, if the Planned Responses were also correct, participants earned double points in that trial. This reward scheme was designed to ensure that participants try to maximize their performance throughout the task. After a general instruction of the task and the required responses, participants were further introduced to the group specific reward conditions. E.g., for the standard-low surprise-high participants the explanation was done as following: ‘If you responded correctly to the second symbol, a # or a &, you will earn 10 points. If you also responded correctly to the first symbol, those points are doubled, so you earn 20 points. No points are earned, if the response to the second symbol was incorrect, regardless of the response to the first symbol.’ Trials with an incorrect Immediate Response were neither rewarded nor analyzed, since correct Immediate Response RTs are necessary to study the binding effect in the R-R task^20,28^.

To examine the effect of reward magnitude, both samples of reward manipulation participants were exposed to different overall reward conditions: standard low—surprise high group could earn 10 or 20 points per trial (low reward condition), and standard high—surprise low group could earn 50 or 100 points per trial (high reward condition). For comparison, a third sample in which participants performed the R-R task without receiving rewards (i.e. no reward group), was formed from a previous study of our work group^28^ and newly recruited subjects.

To investigate surprise effects in the reward manipulation groups, we aimed to evoke schema-discrepancy among those participants^29^. To build schema-discrepancy, first, a schema was established in so-called standard trials, which made up 224 out of the 256 trials in the experiment. In standard trials, stimuli were displayed in white font and corrected responses were rewarded as previously described (see Fig. 1A). The remaining 32 trials (i.e., surprise trials) did break with this schema by displaying stimuli in green color (see Fig. 1B), and by reversing the earnable points, i.e., the magnitude of reward, between both reward groups. For example, participants of the standard low—surprise high group could earn 10 or 20 points in every standard trial, but 50 or 100 points in surprise trials. Therefore, surprise trials meant increase of reward magnitude by a factor of five for standard low—surprise high group (i.e., surprise high trials, switch from low to high reward condition), and a decrease of reward magnitude by a factor of five for standard high—surprise low group (i.e., surprise low trials, switch from high to low reward condition). Combinations of reward conditions and trial conditions, and the associated point values for both reward manipulation groups are shown in Table 1. Following the general and the reward instruction, participants were also introduced to the color-coding and the group-specific change in reward magnitude. E.g., the standard-low surprise-high participants were told: ’Sometimes it will happen that you see the symbols in green color. Please respond to the symbols the same way as usual. The green color means that in those trials, you will earn 50 points for a correct response to the second symbol and 100 points if the response for the first symbol is also correct.’ By presenting surprise trials only 32 times, they were perceived as low probability events; thus, induce surprise effects^12^. Thus, surprise in our study was induced in two ways, combining two types of surprise: by a change of reward magnitude (i.e. unsigned reward prediction error) and by being less probable (i.e. state prediction error)^23,30^. Further in this paper, we will refer to this combination of those two types, simply as “surprise”. To distinguish between two directions of reward magnitude change, the terms “positive surprise” (i.e. raise of reward magnitude) and “negative surprise” (i.e. decrease of reward magnitude) will be used. This is to avoid misconception with the terms positive or negative prediction errors^23,30^, since our design does not separate the reward prediction error from the state prediction error. The color-coding of trials was established to also ensure participants identified the presented trial condition immediately. Thus, surprise effects were evoked with the start of the trial. This design allowed us to independently modulate effects of reward magnitude and surprise.

**Table 1:** Reward conditions in standard and surprise trials for both reward manipulation groups.

	Standard low—surprise high group		Standard high—surprise low group	
	Only immediate response correct	Both responses correct	Only immediate response correct	Both responses correct
Standard trials	Standard low trials		Standard high trials	
Earnable points	10 points	20 points	50 points	100 points
Surprise trials	Surprise high trials		Surprise low trials	
Earnable points	50 points	100 points	10 points	20 points

To distinguish surprise from general environmental conditions (i.e. color change of trials), we conducted an additional, fourth sample of participants, which were rewarded for correct responses and encountered the same color change in the same frequency, but without any change of reward magnitude. Thus, with color being the only difference between trials for this sample, we can ensure that surprise effects found in our experimental groups are actually based on surprise rather than stimulus features.

### Data analysis and statistics

Statistical analyses of the behavioral data were performed by using SPSS. Performances of the Immediate Response reflect action file binding^20,28^, therefore, analyses were run on Stimulus B responses. Mean accuracy (percentage of correct responses) and means of RT (for correct responses) were calculated for each participant, each feature overlap condition (i.e., overlapping and non-overlapping), and each reward condition. To quantify response variability, standard deviations (SDs) of RTs were calculated and investigated using the same experimental factors. Since performance of the Planned Response does not reflect action file binding effects, analyzing those parameters in detail was not the main focus of our analyses. All three responses to Stimulus A belong to one response chain and are therefore reported as done in previous papers regarding the R-R task: as accumulated RT and average accuracy of all three responses^5,20,28^. Action file binding was calculated as a difference of the feature overlap minus the no feature overlap conditions, separately for accuracy, RT and SD_RT_ of the Immediate Response. Higher values indicate stronger binding effects. To investigate effects of reward magnitude (low reward vs high reward) and of surprise (standard trials vs surprise trials), repeated measures ANOVAs with Bonferroni-adjusted post-hoc analysis were used on accuracy, RT, and SD_RT_ data of the reward manipulation samples. Differences between the binding effects of those reward manipulation samples were analyzed in independent t-tests. Additionally, to distinguish between reward magnitude effects and pure reward effects, binding data of the third sample, i.e. no reward group were compared separately to standard low group and to standard high group also through several independent t-tests. The reported *p* values of all t-tests are FDR-corrected. The Bayes factor as BF_10_ is reported to quantify the evidence for the null hypothesis. In the Bayesian t-tests, the default Cauchy prior was used with the scale of 0.707. For interpretation of the reported Bayes factors, we used and suggest to refer to the categorical evaluation according to Jeffreys^31^. A BF_10_ of 1 would be read as no evidence for H1, up to 3 as anecdotal, up to 10 as moderate, up to 30 as strong, up to 100 as very strong, and above 100 as extreme evidence for the H1. In contrast, values beneath 1 and up to 1/3 would be read as anecdotal, up to 1/10 as moderate, up to 1/30 as strong, up to 1/100 as very strong, and smaller than 1/100 as extreme evidence for the H0.

## Results

**Figure 2:**
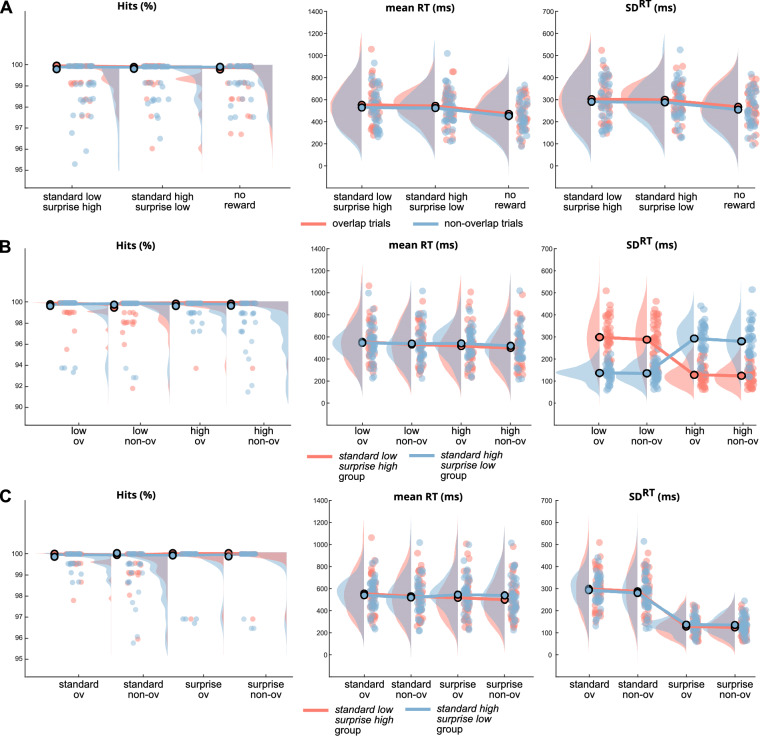
Raincloud scatter plots for accuracy (hits in %), mean RT in ms and standard deviation of RT in ms (SD_RT_) data. The central dot denotes the mean value. (**A**) Comparison of standard low—surprise high, standard high—surprise low and no reward group. The overlap condition is denoted in red color, the non-overlapping condition in blue colors. (**B**) Overlapping versus non-overlapping condition data separated by low and high reward condition. Data for standard low—surprise high group is denoted in red colors, data for standard high—surprise low in blue colors. (**C**) Overlapping versus non-overlapping condition data separated by standard and surprise condition. Data for standard low—surprise high group is denoted in red colors, data for standard high—surprise low in blue colors.

**Figure 3:**
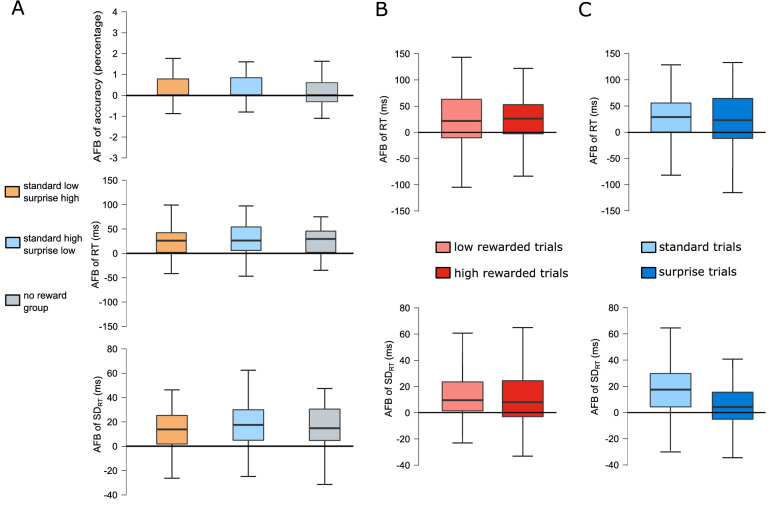
Boxplots depicting action file binding (AFB, i.e., feature overlap minus no feature overlap) for accuracy (hits in %), mean RT in ms and standard deviation of RT in ms (SD_RT_) data. (**A**) Comparison of standard low—surprise high, standard high—surprise low and no reward group for accuracy, RT, and SD_RT_. (**B**) Low (bright red) vs high (dark red) reward condition for RT and SD_RT_ since they showed significant main effects of feature overlap. (**C**) Standard (bright blue) vs surprise (dark blue) condition for RT and SD_RT_ since they showed significant main effects of feature overlap.

To investigate the effects of reward magnitude and surprise on action file binding, we included feedback information about earned points equaling a specific amount of money in a standard action file paradigm. One sample was rewarded low (i.e., with 10 to 20 points) in standard trials (i.e., in 224 of 256 trials) and rewarded high (i.e., with 50 to 100 points) in surprise trials (i.e., in 32 of 256 trials). Another sample was rewarded high (50 to 100 points) in standard trials and rewarded low (10 to 20 points) in surprise trials. This way, conditions and points for both groups were exactly mirror-reversed to one another. For these groups, we first calculated repeated measures ANOVAs on accuracy, response speed (RT), and variability of response speed (SD_RT_) of the Immediate Response. Main and interaction effects of the following factors were analyzed: Feature overlap condition (levels: feature overlap vs. no feature overlap), overall reward magnitude (levels: low vs. high), and surprise (levels: standard trials vs. surprise trials). In these first ANOVA models, we did not consider the group factor (i.e., standard low—surprise high group vs. standard high—surprise low group) because this factor is co-linear with the factors reward magnitude and surprise. To compare binding effects between both reward manipulation groups, we calculated an independent t-test for binding in accuracy, RT, and SD_RT_ later on (see below). Figure 2 shows the distribution and density of the performance data (accuracy, RT, and SD^RT^) across three different groups (Fig. 2A), and across reward and surprise manipulations (Fig. 2B and C). Figure 3 represents action file binding strength (i.e. feature overlap minus no feature overlap) in accuracy, RT and SD_RT_ across three different groups (Fig. 2A), and in RT and SD_RT_ for reward and surprise manipulations (Fig. 3B and C).

### Effects of reward and surprise

The accuracy of the Immediate Response was unaffected by feature overlap (*F*(1,76) = 0.11, *p* = 0.746, *η*^*2*^ = 0.00, *BF*_*10*_ = 0.129), overall reward magnitude (*F*(1,76) = 1.47, *p* = 0.229, *η*^*2*^ = 0.02, *BF*_*10*_ = 0.257), and surprise (*F*(1,76) = 2.06, *p* = 0.155, *η*^*2*^ = 0.03, *BF*_*10*_ = 0.454). Thus, the accuracy parameter showed no binding effect, and remained the same in all overall reward magnitude and surprise manipulations.

Regarding the response speed in trials with correct Immediate Response, a significant effect of feature overlap was found (*F*(1,76) = 8.72, *p* = 0.004, *η*^*2*^ = 0.10, *BF*_*10*_ > 100) with slower response times for the feature overlap (540.31 ms ± 18.58) than for the no feature overlap trials (523.32 ms ± 18.92, *p* = 0.004). Additionally, the RT data showed significant main effects of overall reward magnitude (*F*(1,76) = 26.81, *p* < 0.001, *η*^*2*^ = 0.03, *BF*_*10*_ > 100), and surprise (*F*(1,76) = 6.35, *p* = 0.014, *η*^*2*^ = 0.08, *BF*_*10*_ = 0.614). That is, responses were slower when expecting a low reward (543.56 ms ± 18.88) than a high reward (520.06 ms ± 18.45, *p* < 0.001), and slower in standard trials (537.53 ms ± 18.25) than in surprise trials (526.09 ms ± 19.08, *p* = 0.014). Therefore, a high magnitude of reward and a surprise improves response speed. Regarding binding, neither overall reward magnitude (*F*(1,76) = 0.36, *p* = 0.550, *η*^*2*^ = 0.01, *BF*_*10*_ = 0.143), nor surprise (*F*(1,76) = 2.49, *p* = 0.118, *η*^*2*^ = 0.03, *BF*_*10*_ = 0.377) showed significant interaction with feature overlap.

Similarly to the RT data, a significant main effect of feature overlap (*F*(1,76) = 10.67, *p* = 0.002, *η*^*2*^ = 0.12, *BF*_*10*_ = 1.115) was found for the variability of response speed (i.e. SD_RT_). That is, participants showed a higher variability of response speed in the feature overlap condition (214.20 ms ± 6.67), compared to the no feature overlap condition (206.88 ms ± 7.21, *p* = 0.002). The SD_RT_ was not affected by overall reward magnitude (*F*(1,76) = 2.57, *p* = 0.113, η^2^ = 0.03, *BF*_*10*_ = 0.159), but showed significant effects of surprise (*F*(1,76) = 971.96, *p* < 0.001, *η*^*2*^ = 0.93, *BF*_*10*_ > 100): In standard trials, the variability of response speed was more than twice as high (289.76 ms ± 9.28) as in surprise trials (131.31 ms ± 4.56, *p* < 0.001). Furthermore, a significant interaction of surprise and feature overlap was found (*F*(1,76) = 10.86, *p* = 0.001, *η*^*2*^ = 0.13, *BF*_*10*_ = 4.422). The Bonferroni adjusted post-hoc test showed significant differences between feature overlap conditions only in standard trials (mean difference: 11.94 ms ± 3.11, *p* < 0.001) and not in surprise trials (mean difference: 2.71 ms ± 2.08, *p* = 0.197).

To verify that these found surprise effects in RT and SD_RT_ are indeed due to surprise (and not due to the color change between trials), we performed a RM-ANOVA on the fourth sample with the factors feature overlap and trial color (white trials correspond to the standard trials and green trials to the surprise trials). For RT, a significant effect of feature overlap was found (*F*(1,15) = 1.556, *p* = 0.039, *η*^*2*^ = 0.04, *BF*_*10*_ = 1.391), with higher response times for the feature overlap (543.91 ms ± 17.19) than for the no feature overlap trials (529.25 ms ± 16.44, *p* = 0.039). In contrast to surprise, trial color had no significant effect on response times (*F*(1,15) = 1.374, *p* = 0.259, *η*^*2*^ = 0.017, *BF*_*10*_ = 0.320). For SD_RT_, again, a significant main effect of feature overlap (*F*(1,15) = 1.303, *p* = 0.048, *η*^*2*^ = 0.001, *BF*_*10*_ = 0.407) was found. That is, participants showed a higher variability of response speed in the feature overlap condition (215.40 ms ± 6.37), compared to the no feature overlap condition (209.5 ms ± 6.89, *p* = 0.048). The SD_RT_ showed a significant main effect of trial color (*F(1,15)* = 145.5, *p* < 0.001, *η*^*2*^ = 0.884, *BF*_*10*_ > 100) equal to the main effect of surprise: In white trials (equally to standard trials), the variability of response speed was twice as high (289.71 ms ± 22.66) as in green trials (equally to surprise trials, 135.19 ms ± 10.40, *p* < 0.001). However, no significant interaction of trial color and feature overlap was found (*F(1,15)* = 0.061*, p* = 0.808, *η*^*2*^ = 0.001, *BF*_*10*_ = 0.419). In conclusion, the found interaction with action file binding in standard low surprise high group and standard high surprise low group is actually based on surprise and not on color differences of trials.

Based on these results, we also conducted a Bonferroni adjusted post-hoc test for the non-significant interaction between feature overlap and surprise on RT. In contrast to the SD_RT_, the RT post-hoc analysis showed both, a significant difference between feature overlap conditions in standard trials (mean difference: 22.29 ms ± 6.63, *p* = 0.006), and in surprise trials (mean difference: 16.74 ms ± 6.22, *p* = 0.048).

The difference between feature overlap RT and no feature overlap RT represents action file binding strength. Thus, the difference between variability of feature overlap RT (i.e. feature overlap SD_RT_) and no feature overlap RT (i.e. no feature overlap SD_RT_) presumably reflects the variability of action file binding strength. Consequently, the data suggest that surprise decreases variability of action file binding strength, but not the mean magnitude of binding. Reward magnitude had no effect on variability or the mean magnitude of binding.

### Comparison of effects between reward manipulation groups

To compare binding effects between both reward manipulation groups, we calculated an independent t-test for the binding effect (i.e., difference feature overlap minus no feature overlap) in accuracy, RT, and SD_RT_. In general, no significant differences in accuracy were found (*t*(76) = 0.682, *p* = 0.497, d = 0.154, *BF*_*10*_ = *0.287*). The same was the case for the RT (*t*(76) = 0.396, *p* = 0.693, d = 0.09, *BF*_*10*_ = *0.251*) and the SD_RT_ (*t*(76) = 0.396, *p* = 0.903, d = 0.028, *BF*_*10*_ = *0.236*). Standard low trials and standard high trials did also not differ in accuracy (*t*(76) = 0.552, *p* = 0.583, d = 0.125, *BF*_*10*_ = *0.268*), RT (*t*(76) = 0.208, *p* = 0.836, d = 0.047, *BF*_*10*_ = *0.239*), or SD_RT_ (*t*(76) = 0.218, *p* = 0.828, d =  − 0.049, *BF*_*10*_ = *0.240*). Finally, no significant differences between surprise high trials and surprise low trials occurred in accuracy (*t*(58.124) = 0.916, *p* = 0.362, d = 0.207, *BF*_*10*_ = *0.338*), RT (*t*(76) = 0.717, *p* = 0.476, d = 0.162, *BF*_*10*_ = *0.294*), or SD_RT_ (*t*(76) = 0.591, *p* = 0.556, d = 0.134, *BF*_*10*_ = *0.273*). Thus, the factor group did not affect binding effects. As mentioned before, group membership reflects the combination of overall reward magnitude and surprise: standard low—surprise high group experienced surprise as positive (i.e. reward magnitude increases in surprise trials), while standard high—surprise low group experienced surprise as negative (i.e. reward magnitude decreases in surprise trials). Since no significant differences in the measured binding effects was found between the groups, this indicates that surprise influences binding strength regardless of its direction (i.e., positive or negative surprise).

### Comparison to the group receiving no rewards

To distinguish between reward magnitude effects and surprise effects on action file binding, we performed t-tests comparing a third control sample (i.e., a no reward group) and each reward manipulation group. The control group performed the original R-R task without reward magnitude and surprise manipulations. We compared the binding effects (i.e. difference feature overlap minus no feature overlap) in all trials of no reward group to binding effects of the reward manipulation groups in general (i.e. in all trials, regardless of reward magnitude and surprise) and in all four trial conditions (i.e. in standard low trials, standard high trials, surprise low trials and in surprise high trials).

Comparing binding effects, only the accuracy binding of standard low—surprise high group was significantly higher (0.38 ± 1.36) than binding of no reward group (− 0.36 ± 1.15), (*t*(71) = 2.519,* p* = 0.028* d* = 0.591, *BF*_*10*_ = *0.778*). Similarly, accuracy binding in standard low trials were significantly higher (0.44 ± 1.50) in comparison to no reward group (− 0.36 ± 1.15), (*t*(71) = 2.538,* p* = 0.028,* d* = 0.596, *BF*_*10*_ = *1.473*). In our repeated measures ANOVA of the reward manipulation groups, we found that binding in SD_RT_ is vastly higher in both kinds of standard trials than in both kinds of surprise trials. Therefore, the comparison between binding in both surprise trials of the reward manipulation groups and the no reward group was particularly interesting. Indeed, the SD_RT_ binding in surprise high trials (3.94 ± 15.02) and in surprise low trials (1.48 ± 21.20) was significantly lower than in all trials of *no reward group* (12.24 ± 18.99), (*t*(71) =  − 2.084,* p* = 0.041,* d* =  − 0.489, *BF*_*10*_ = *2.283*; *t*(71) =  − 2.270,* p* = 0.035,* d* =  − 0.533, *BF*_*10*_ = *2.957*). None of the other comparisons between *no reward group* and the reward manipulation groups were significant (*p* ≥ 0.134, *BF*_*10*_ ≤ *0.316*). Thus, surprise, regardless of it being positive (i.e., increasing reward magnitude) or negative (i.e., decreasing reward magnitude), decreases variability of action file binding strength.

## Discussion

The goal of the current study was to provide further insights into the nature of bindings between different motor features constituting complex motor sequences. To this end we examined in how far motor feature bindings are affected by reward magnitude or the effects of an unsigned surprise signal. As an experimental approach, we used an established paradigm inspired by the theory of event coding framework^1,18^—an action file paradigm. The Theory of Event Coding assumes that stimulus features and action features that underly perception and action planning, are coded and stored together^1^. This action file paradigm was modified by adding reward magnitude and surprise manipulations. This was done in two orthogonal manipulations of reward magnitude and surprisingness of the reward in two samples of participants. A third sample of participants performing the R-R task in its original version, was used as a control group for reward and surprise manipulations. A fourth sample performed a version similar to the first two samples, but without a change of reward manipulations, to control for the impact of general experimental features (i.e. color change between trials) in surprise effects.

We found that the consistency of action file binding strength is modulated by surprise, but not by reward magnitude. Surprise decreases variability of action file binding strength. Importantly, the quality of the surprise was not essential for this effect since RT variability modulations did not differ depending on whether the surprise was positive (increase of reward magnitude) or negative (decrease of reward magnitude). For reward magnitude and surprise modulation groups, both qualities of surprise were indicated by the stimuli color at the beginning of trials (white for standard reward, green for increase or decrease of reward magnitude). We were able to rule out the possibility that the mere change of color had caused this effect, since the sample which did not experience any change of reward magnitude (i.e. no positive or negative surprise) when trial colors changed, did not show a decreased variability of action file binding strength. Opposed to the consistency of action file binding strength across trials, the average magnitude of action file binding effects was not modulated by reward or surprise manipulations. A more fine-grained interpretation is that surprise signals recruit an attentional orienting response reducing time-on-task effects such as attentional lapses or mind-wandering, and thereby reducing variability in processing leading in turn to less variability in action file binding effects. In other words, the surprise signals may indicate to participants that the upcoming trial is "special", thereby allocating more task-directed attention and as a consequence more consistent behavior emerges as in the other trials which are likely subject to occasional attentional lapses. Such effects of decreasing behavioral variability via attention allocation is consistent with other findings reported in the literature^32,33^. Taken together, surprise might not directly influence the volatility of action file binding but might do so via attention allocation^34^.

The used established R-R paradigm has no time limit for Planned and Immediate Response, which can encourage to sacrifice faster reaction time in favor of a higher hit rate (i.e. speed-accuracy trade-off)^5,35^. With such high accuracy scores as observed in this paradigm, it is possible that a significant main effect of feature overlap (i.e. action file binding) on accuracy is masked. Nevertheless, there is no indication that action file binding strength in accuracy would be affected by surprise, since action file binding strength in response speed was not modulated by surprise either. A modification of the classical R-R paradigm with an increased degree of difficulty, causing lower accuracy scores, could further secure our conclusion on the role of unsigned surprise on motor feature integration.

Several theoretical frameworks have been put forward to conceptualize the effects of unsigned surprise signals^12^. One framework, the so-called predicted response outcome (PRO) model, suggests that medial prefrontal brain structures (especially the anterior cingulate cortex, ACC) predict the likely outcome of action un-related to the valence of that outcome. This theory’s aim is to clarify how we make predictions about potential outcomes before carrying out an action, as well as how we evaluate afterwards the degree in which our predictions match the actual outcomes^36^. According to the PRO model, only the unexpectedness (surprisingness) is essential for action control. The current findings on the modulability of action file bindings are in line with that model; however, it was not average binding strength that was altered, but the consistency in binding effects. This outcome is not directly predicted by the PRO model of unsigned surprise signaling, but can nevertheless be explained by the PRO model. Regarding this, it is important to consider that the PRO model is built on assumptions of the reinforcement learning theory and temporal difference learning model in particular^12^. Viewed from a neurobiological perspective, computational principles of the models are related to the functioning of the dopaminergic system, making it likely that also the dopaminergic system is triggered by surprise signals^37,38^. Crucially, the dopaminergic system is not only implicated in the processing of rewards and surprise signals. Rather, the dopaminergic system does also modulates the consistency of (neural) processes underlying cognitive functions^27^. The likely reason is that increased dopaminergic activity reflects the recruitment of attention as much as it decreases neuronal noise^26,39^, which leads to more distinct and stable cortical representations resulting in decreases and variability in cognitive performance^27^. These effects have also been referred to as gain control, which describes a general working principle in neural networks found at sensory, cognitive^40–42^ and motor levels^43,44^. Increasing gain control can be viewed as sharpening the responsivity of the neural network and as increasing the signal–noise-ratio (SNR) of information processing. Byres and Serences^45^ note that mechanisms aimed to increase top-down (cognitive) control and processes subserving learning-relevant mechanisms (such as surprise processing) operate by increasing the SNR in neural circuits^46^. This increase in the SNR is then likely leading to a higher consistency in binding and motor feature integration effects as observed in the current study.

However, considerations related to principles of neural gain control have not been much around in the Theory of Event Coding^47^, because this theory is mostly ground in cognitive psychology. Nevertheless, propositions of neural gain control are commensurable with the theoretical framing of TEC. This becomes particularly clear when considering the so-called “meta-control state model (MSM)”^48^, closely connected to TEC. The dopaminergic system is known to affect metacontrol processes^49–51^. In recent years, TEC has been connected to so-called metacontrol frameworks^49,52–54^ describing that response selection (and cognitive control processes) are not static. According to the metacontrol hypothesis^49^ there is evidence suggesting that cognitive control is not a unitary function but an emerging property of the interaction of systems promoting cognitive persistence including focusing on one goal and on relevant information, and systems promoting cognitive flexibility that is needed, for instance, for switching to other plans, opening up for other opportunities, and considering a broader range of possibilities. Following the MSM, a person can be biased towards persistence or towards flexibility, when two or more goal-directed representations compete for selection^48,49^. Recent findings from our research group showed that a bias towards cognitive persistence reduces binding effects^55^. This is the case because more top-down control sustains an effective focus on one goal and on relevant information. Thus, not goal-compatible information is discarded reducing time-consuming binding reconfiguration processes. The current results suggest that this is also associated with an increase in the temporal consistency of binding reconfiguration processes taking place. The results obtained are thus compatible with several theoretical conceptions, for which the current results also suggest that these can be integrated.

## Data Availability

The data underlying this article will be shared on reasonable request to the corresponding author.
